# PRMT5 Is Upregulated in HTLV-1-Mediated T-Cell Transformation and Selective Inhibition Alters Viral Gene Expression and Infected Cell Survival

**DOI:** 10.3390/v8010007

**Published:** 2015-12-30

**Authors:** Amanda R. Panfil, Jacob Al-Saleem, Cory M. Howard, Jessica M. Mates, Jesse J. Kwiek, Robert A. Baiocchi, Patrick L. Green

**Affiliations:** 1 Center for Retrovirus Research, The Ohio State University, Columbus, OH 43210, USA; panfil.6@osu.edu (A.R.P.); al-saleem.1@osu.edu (J.A.-S.); howard.937@osu.edu (C.M.H.); kwiek.2@osu.edu (J.J.K.); 2 Department of Veterinary Biosciences, The Ohio State University, Columbus, OH 43210, USA; 3 Center for Microbial Interface Biology, The Ohio State University, Columbus, OH 43210, USA; mates.6@osu.edu; 4 Department of Microbiology and Department of Microbial Infection and Immunity, The Ohio State University, Columbus, OH 43210, USA; 5 Division of Hematology, Department of Internal Medicine, The Ohio State University, Columbus, OH 43210, USA; baiocchi.1@osu.edu; 6 Comprehensive Cancer Center and Solove Research Institute, The Ohio State University, Columbus, OH 43210, USA

**Keywords:** HTLV-1, PRMT5, transformation, ATLL, Tax, HBZ, p30, lymphoma

## Abstract

Human T-cell leukemia virus type-1 (HTLV-1) is a tumorigenic retrovirus responsible for development of adult T-cell leukemia/lymphoma (ATLL). This disease manifests after a long clinical latency period of up to 2–3 decades. Two viral gene products, Tax and HBZ, have transforming properties and play a role in the pathogenic process. Genetic and epigenetic cellular changes also occur in HTLV-1-infected cells, which contribute to transformation and disease development. However, the role of cellular factors in transformation is not completely understood. Herein, we examined the role of protein arginine methyltransferase 5 (PRMT5) on HTLV-1-mediated cellular transformation and viral gene expression. We found PRMT5 expression was upregulated during HTLV-1-mediated T-cell transformation, as well as in established lymphocytic leukemia/lymphoma cell lines and ATLL patient PBMCs. shRNA-mediated reduction in PRMT5 protein levels or its inhibition by a small molecule inhibitor (PRMT5i) in HTLV-1-infected lymphocytes resulted in increased viral gene expression and decreased cellular proliferation. PRMT5i also had selective toxicity in HTLV-1-transformed T-cells. Finally, we demonstrated that PRMT5 and the HTLV-1 p30 protein had an additive inhibitory effect on HTLV-1 gene expression. Our study provides evidence for PRMT5 as a host cell factor important in HTLV-1-mediated T-cell transformation, and a potential target for ATLL treatment.

## 1. Introduction

Human T-cell leukemia virus type-1 (HTLV-1) is a tumorigenic retrovirus that infects an estimated 15–20 million people worldwide [1]. This blood-borne pathogen is the causative infectious agent of adult T-cell leukemia/lymphoma (ATLL), a disease of CD4+ T-cells [2,3,4]. HTLV-1 is also associated with inflammatory disorders such as HTLV-1-associated myelopathy/tropical spastic paraparesis (HAM/TSP) [5,6]. The likelihood of developing ATLL is between 2%–6% during the lifetime of an infected individual [7], with symptoms taking up to 20–30 years to present. Despite the long clinical latency period, diseases such as ATLL are extremely aggressive and usually fatal. ATLL is highly chemotherapy-resistant, and while many current therapies (e.g., antivirals AZT/IFN-α, proteasome inhibitors, anti-CCR4 monoclonal antibody) improve ATLL patient survival (reviewed in [8]), the patients consistently relapse.

As a complex retrovirus, HTLV-1 has a genome that encodes structural and enzymatic proteins (Gag, Pro, Pol, Env), regulatory proteins (Tax and Rex), and several accessory proteins (p30, p12/p8, HBZ). Studies have shown that at least two viral gene products, Tax and HBZ, have transforming properties and play a role in the pathogenic process [9,10]. Tax acts as a viral transcriptional activator of HTLV-1 gene expression through activation of the viral long terminal repeat (LTR) and various cellular signaling pathways such as CREB, NF-κB, and AP-1 [11,12]. Tax also causes deregulation of the cell cycle by silencing cellular checkpoints that guard against DNA structural damage and abnormal chromosomal segregation, thus leading to the accumulation of mutations in HTLV-1 infected cells [13]. However, Tax expression is lost in greater than 70% of ATLL cells due to genetic and/or epigenetic changes in the HTLV-1 provirus, which include deletion or methylation of the viral 5' LTR. These changes abolish expression of other viral genes with the exception of HBZ. In fact, HBZ is the only viral gene that is intact and expressed in all ATLL cases [14,15]. HBZ protein is expressed from a promoter located in the viral 3' LTR; current data indicates that HBZ promotes proliferation of ATLL cells through both its mRNA and protein forms [15,16]. HBZ protein has also been shown to interact with several cellular transcription factors such as CREB and CBP, p300, JunD, JunB, and c-Jun and to act as a negative regulator of Tax-mediated HTLV-1 transcription [17,18,19,20,21,22].

Although Tax is indispensable for viral transformation, the mechanisms by which the virus persists *in vivo* and transforms CD4+ T-cells are not completely understood. The requirement for Tax and other viral proteins *in vivo* suggests that expression of viral proteins early in infection plays a major role in viral replication, infected cell survival, and disease development. A favored theory within the field is that the virus is critically dependent on Tax early in infection to initiate transformation, but then Tax expression is highly regulated and often times silenced to prevent immune detection. HBZ is hypothesized to provide the maintenance or cell survival signal necessary for the transformation process. Over time, the combination of genetic and epigenetic changes in an HTLV-1-infected cell can lead to transformation and potentially, disease development [23]. While we know that the viral proteins Tax and HBZ are intimately involved in the cell transformation process, neither is sufficient, which suggests the involvement of cellular factors.

Chromatin remodeling complexes and associated co-repressors such as histone deacetylases (HDAC), DNA methyltransferases (DNMT), and protein arginine methyltransferase 5 (PRMT5) participate in silencing tumor suppressor gene expression and contribute to cellular transformation [24,25,26]. Recent reports have indicated PRMT5 over-expression as relevant to the pathogenesis of many cancers, including lymphomas, melanomas, and astrocytomas [27,28,29,30,31,32]. PRMT5 is a type II PRMT enzyme that silences the transcription of key regulatory genes by symmetric di-methylation (S2Me) of arginine (R) residues on histone proteins (H4R3 and H3R8) [33]. PRMT5 is also involved in a wide variety of cellular processes, including RNA processing, transcriptional regulation, and signal transduction pathway regulation that are highly relevant to the pathogenesis of cancer [34,35,36]. Recently, PRMT5 was found to play a critical role in Epstein-Barr virus (EBV)—driven B-cell transformation [31].

Our group previously identified PRMT5 as a binding partner of the HTLV-1 accessory protein p30, using mass spectrometry [37]. p30 is encoded from a doubly spliced mRNA and is dispensable for viral infection and T-cell transformation *in vitro*, but is required for establishment of viral persistence in an *in vivo* rabbit model of infection [38,39]. p30 negatively regulates viral gene transcription at both the transcriptional and post-transcriptional levels by competing with Tax for binding to CBP/p300 and retaining the *tax/rex* mRNA in the nucleus, respectively [40,41,42].

Currently, there have been no studies investigating the role of PRMT5 in T-cell malignancies, including HTLV-1-associated disease. Therefore, we sought to determine if PRMT5 plays a role in HTLV-1 transformation/malignancy. Indeed, we found PRMT5 levels were upregulated during T-cell transformation and in established lymphocytic leukemia/lymphoma cell lines. Our data suggested that PRMT5 negatively regulated HTLV-1 viral gene expression, which indicated that PRMT5 could be an important cellular regulator of the viral transformation process. Furthermore, selective inhibition of PRMT5 by a novel small molecule inhibitor (PRMT5i) in HTLV-1-positive cell lines reduced cell survival; therefore, PRMT5 may represent an important therapeutic target for ATLL.

## 2. Materials and Methods

### 2.1. Cell Lines and Culture

HEK293T and pA-18G-BHK-21 cells were maintained in Dulbecco’s modified Eagle’s medium (DMEM) supplemented with 10% fetal bovine serum (FBS) (Gemini Bio-Products, Broderick, CA, USA), 2 mM glutamine, penicillin (100 U/mL), and streptomycin (100 μg/mL). PBL-ACH and ACH.2 cells (early passage HTLV-1-immortalized human T-cells) were maintained in RPMI 1640 supplemented with 20% FBS, 10 U/mL recombinant human interleukin-2 (rhIL-2; Roche Applied Biosciences, Indianapolis, IN, USA), 2 mM glutamine, 100 U/mL penicillin, and 100 μg/mL streptomycin. SLB-1 cells (HTLV-1-transformed T-cell line) were maintained in Iscove’s medium supplemented with 10% FBS, 2 mM glutamine, 100 U/mL penicillin, and 100 μg/mL streptomycin. C8166, MT-1, MT-2, Hut-102 (HTLV-1-transformed T-cell lines), Hut-78 and Jurkat cells (HTLV-1-negative transformed T-cell lines) were maintained in RPMI 1640 supplemented with 10% FBS, 2 mM glutamine, 100 U/mL penicillin, and 100 μg/mL streptomycin. TL-Om1, ATL-43T, and ATL-ED cells (ATL-derived T-cell lines) were maintained in RPMI 1640 supplemented with 10% FBS, 2 mM glutamine, 100 U/mL penicillin, and 100 μg/mL streptomycin. ATL-55T cells (ATL-derived T-cell line) were maintained in RPMI 1640 supplemented with 20% FBS, 20 U/mL rhIL-2, 2 mM glutamine, 100 U/mL penicillin, and 100 μg/mL streptomycin. The parental 729.B (uninfected) and derivative 729.ACH (HTLV-1 producing) cell lines were maintained in Iscove’s medium supplemented with 10% FBS, 2 mM glutamine, 100 U/mL penicillin, and 100 μg/mL streptomycin. ACH-2 (HIV-1_LAV_) cells were obtained through the AIDS Research and Reference Reagent Program, Division of AIDS, NIAID, NIH. ACH-2 cells (HIV-1_LAV_) were maintained in RPMI 1640 supplemented with 10% FBS, 2 mM glutamine, 100 U/mL penicillin, and 100 μg/mL streptomycin. All cells were grown at 37 °C in a humidified atmosphere of 5% CO_2_ and air. The SeAx cell line was derived from peripheral blood of a patient with Sezary syndrome (kind gift from Dr. Henry Wong, The Ohio State University). Human PBMCs were isolated using Ficoll-Paque PLUS (GE Healthcare Life Sciences, Pittsburgh, PA, USA) and naïve T-cells were enriched using a Pan T-Cell Isolation Kit (Miltenyi Biotec, Inc., Gaithersburg, MD, USA). Whole blood samples obtained from ATLL patients were a generous gift from Dr. Lee Ratner (Washington University, St. Louis, MO, USA).

### 2.2. Plasmids and Cloning

Plasmid DNA was purified on maxi-prep columns according to the manufacturer’s protocol (Qiagen, Valencia, CA, USA). The flag-tagged PRMT5 expression vector was described previously [37]. p30 cDNA was cloned into the pcDNA3.1 expression vector (Invitrogen, Grand Island, NY, USA) to create pcDNA3.1-p30. The S-tagged Tax and HBZ expression vectors contained Tax or HBZ cDNA inserted into a pTriEx™-4 Neo vector (Novagen, Madison, WI, USA) for mammalian cell expression of S-tagged Tax and HBZ proteins. The plasmid containing the wild-type HTLV-1 infectious proviral clone, ACHneo, was described previously [43]. The LTR-1-luciferase reporter plasmid and transfection efficiency control plasmid TK-renilla were described previously [41].

### 2.3. Immunoblotting

Cell lysates from luciferase assays were harvested in Passive Lysis Buffer (Promega, Madison, WI, USA) containing protease inhibitor cocktail (Roche) and quantitated using an ND-1000 Nanodrop spectrophotometer (ThermoFisher, Waltham, MA, USA). All other cell lysates were harvested in NP-40 lysis buffer containing protease inhibitor cocktail, and were quantitated using the Pierce^TM^ BCA Protein Assay Kit (ThermoFisher) and a FilterMax F5 Multi-Mode Microplate Reader (Molecular Devices, Sunnyvale, CA, USA). Equivalent amounts of protein were separated in Mini-PROTEAN^®^ TGX™ Precast 4%–20% Gels (Bio-Rad, Hercules, CA, USA) and transferred to nitrocellulose membranes. Membranes were blocked in PBS containing 5% milk and 0.1% Tween-20 and incubated with primary antibody. The following antibodies were used: anti-PRMT5 (ab31751, 1:1000; Abcam, Cambridge, MA, USA), anti-p19 (patient anti-sera specific for gag proteins), anti-Flag clone M2 (1:1000; Agilent, Wilmington, DE, USA), anti-p30 (rabbit anti-sera), anti-Tax (rabbit anti-sera), anti-HBZ (rabbit anti-sera), anti-p27 (1:250; Santa Cruz Biotechnology, Dallas, TX, USA), anti-p21 (1:250; Santa Cruz Biotechnology), anti-cyclin B1 (1:250; Santa Cruz Biotechnology), and anti-β-actin 1:5000 (Sigma, St. Louis, MO, USA). The secondary antibodies used were HRP goat-anti-rabbit and goat-anti-mouse (1:5000; Santa Cruz Biotechnology). Blots were developed using Immunocruz Luminol Reagent (Santa Cruz Biotechnology). Images were taken using the Amersham Imager 600 (GE Healthcare Life Sciences) and densitometric data was calculated using the ImageQuantTL program (GE Healthcare Life Sciences).

### 2.4. Quantitative RT-PCR

Total RNA was isolated from 10^6^ cells per condition using the RNeasy Mini Kit (Qiagen) according to the manufacturer’s instructions. Isolated RNA was quantitated and DNase-treated using recombinant DNase I (Roche). Reverse transcription was performed using the Omniscript RT Kit (Qiagen) according to the manufacturer’s instructions. The instrumentation and general principles of the CFX96 Touch™ Real-Time PCR Detection System (Bio-Rad) are described in detail in the operator’s manual. PCR amplification was carried out in 96-well plates with optical caps. The final reaction volume was 20 μL consisting of 10 μL iQ™ SYBR^®^ Green Supermix (Bio-Rad), 300 nM of each specific primer, and 2 μL of cDNA template. For each run, sample cDNA and a no-template control were assayed in triplicate. The reaction conditions were 95 °C for 5 min, followed by 40 cycles of 94 °C for 30 s, 56 °C for 30 s, and 72 °C for 45 s. Primer pairs to specifically detect viral mRNA species (tax, hbz), prmt5, st7, and gapdh were described previously [28,44]. Data are presented in histogram form as means with standard deviations from triplicate experiments.

### 2.5. Co-Culture Immortalization Assays

Long-term immortalization assays were performed as detailed previously [45]. Briefly, 2 × 10^6^ freshly isolated human PBMCs were co-cultivated at a 2:1 ratio with lethally irradiated cells (729.B uninfected parental; 729.ACH HTLV-1-producing) in 24-well culture plates (media was supplemented with 10 U/mL rhIL-2). HTLV-1 gene expression was confirmed by the detection of p19 Gag protein in the culture supernatant, and was measured weekly by p19 ELISA (Zeptometrix, Buffalo, NY, USA). Viable cells were counted weekly by Trypan blue dye exclusion.

### 2.6. Packaging and Infection of Lentivirus Vectors

Lentiviral vectors expressing five different PRMT5-directed shRNAs (target set RHS4533-EG10419), and the universal negative control, pLKO.1 (RHS4080) were purchased from Open Biosystems (Dharmacon, Lafayette, CO, USA) and propagated according to the manufacturer’s instructions. HEK293T cells were transfected with lentiviral vector(s) expressing shRNAs, plus DNA vectors encoding HIV Gag/Pol and VSV-G in 10-cm dishes with Lipofectamine^®^2000 according to the manufacturer’s instructions. Media containing lentiviral particles was collected 72 h later and filtered through 0.45 µm pore size filters. Lentiviral particles were then concentrated using ultracentrifugation in a Sorvall SW-41 swinging bucket rotor. Lymphoid cell lines were infected with the concentrated lentivirus in 8 µg/mL polybrene by spinoculation at 2000 x*g* for 2 h at room temperature. HEK293T cells were infected with the concentrated lentivirus in 8 μg/mL polybrene. After 72 h, stable cell lines were selected by treatment with 1–2 µg/mL puromycin for 7 days.

### 2.7. PRMT5i Treatment

A selective PRMT5 inhibitor (PRMT5i) drug was recently described by Alinari *et al.* [31]. Lymphoid cells were seeded into a 12-well plate at 0.5 × 10^6^ cells/mL. Indicated concentrations of the PRMT5i were added to duplicate wells. Cells were incubated at 37 °C for 48 h. After incubation, cell viability and proliferation were measured. Cell viability was determined using Trypan blue dye exclusion. Cellular proliferation was measured in duplicate in each condition using the CellTiter 96^®^ AQ_ueous_ One Solution Cell Proliferation Assay (Promega). Cells were collected by slow centrifugation (5 min, 800 x*g*) for downstream qRT-PCR analysis as described above.

### 2.8. HIV-1 Gene Expression in Chronically Infected Cells

ACH-2 (HIV-1_LAV_) cells were seeded into a 12-well plate at 1.6 × 10^6^ cells/mL. Indicated concentrations of PRMT5i were added in triplicate wells. Cells were incubated at 37 °C for 48 h. After incubation, 10 µL of culture supernatant was removed and freeze-thawed once for reverse transcriptase (RT) assays. Briefly, 10 µL of culture supernatant was incubated overnight at 37 °C with 25 µL buffer (50 mM Tris-HCl pH 7.8, 75 mM KCl, 2 mM DTT, 5 mM MgCl_2_, 5 µg/mL Poly_(dA:dT)_, and 0.5% NP-40) containing 10 µCi/mL [α^32^P]-labeled dTTP (Perkin Elmer, Waltham, MA, USA). A volume of 10 µL was spotted onto a DEAE filtermat (Perkin Elmer), air dried at room temperature, then washed 5× with 1× saline-sodium citrate buffer (SSC) and 2× with 85% ethanol. Filtermats were air dried and exposed to a phosphorimaging screen for 2.5 h at room temperature. Density, counts/mm^2^, was determined using the Typhoon Scanner (GE Healthcare Life Sciences) and Quantity One software (Bio-Rad).

### 2.9. Transient Transfections, Reporter Assays, and p19 Gag ELISA

HEK293T cells were transfected using Lipofectamine^®^2000 Transfection Reagent according to the manufacturer’s instructions. Each transfection experiment was performed in triplicate and presented as means plus standard deviations. In general, HEK293T cells in a 6-well dish were transfected with approximately 1–2 μg total DNA consisting of 20 ng TK-renilla (transfection control), 100 ng LTR-1-luciferase, 1 μg ACHneo, 500 ng Flag-PRMT5, 500 ng pcDNA3.1-p30, 100 ng S-tag-Tax, or 500 ng S-tag-HBZ, where indicated. HEK293T cells were harvested 48 h post-transfection in Passive Lysis Buffer (Promega). Relative firefly and Renilla luciferase units were measured in a FilterMax F5 Multi-Mode Microplate Reader using the Dual-Luciferase^®^ Reporter Assay System (Promega) according to the manufacturer’s instructions. Each condition was performed in duplicate. Extracts also were subjected to immunoblotting to verify equivalent protein levels. Cell supernatants (48 h) were used for p19 ELISA (Zeptometrix).

### 2.10. Annexin V Staining

Lymphoid cells were seeded into a 6-well plate at 1 × 10^6^ cells/mL. Indicated concentrations of the PRMT5i were added to the appropriate wells. Cells were incubated at 37 °C for 24 h. After incubation, cells were collected by slow centrifugation (5 min, 800 x*g*) for apoptosis analysis via flow cytometry. Collected cells were stained using the FITC Annexin V Apoptosis Detection Kit I (BD Biosciences; San Diego, CA, USA) according to the manufacturer’s instructions.

### 2.11. ChIP Assays

pA-18G-BHK-21 cells are a Syrian Hamster kidney cell line stably transfected with a plasmid vector containing the lacZ bacterial gene under the control of a HTLV-1-LTR promoter as previously described [46]. pA-18G-BHK-21 cells were transfected in 10-cm dishes (1 μg ACHneo and 5 μg flag-PRMT5) using Lipofectamine^®^2000 according to the manufacturer’s instructions. Cells were cross-linked in fresh 1% paraformaldehyde for 10 min at room temperature. The cross-linking reaction was quenched using 125 mM glycine. Following cell lysis and DNA fragmentation by sonication, DNA-protein complexes were immunoprecipitated with anti-PRMT5 (Santa Cruz) and control anti-IgG (Santa Cruz) antibodies. Immunoprecipitated DNA-protein complexes were washed using sequential low-salt, high-salt, lithium chloride, and Tris-EDTA (TE) buffers. DNA was purified using the Qiagen Gel Extraction Kit (Qiagen). The presence of specific DNA fragments in each precipitate was detected using PCR. Primers used for amplifying the HTLV-1 LTR were 5'-CCACAGGCGGGAGGCGGCAGAA-3' and 5'-TCATAAGCTCAGACCTCCGGGAAG-3' and LacZ-coding region were 5'-AAAATGGTCTGCTGCTG-3' and 5'-TGGCTTCATCCACCACA-3'. Quantification of each ChIP experiment was performed using ImageJ software.

## 3. Results

### 3.1. PRMT5 Was Upregulated in T-Cell Leukemia/Lymphoma Cells

**Figure 1:**
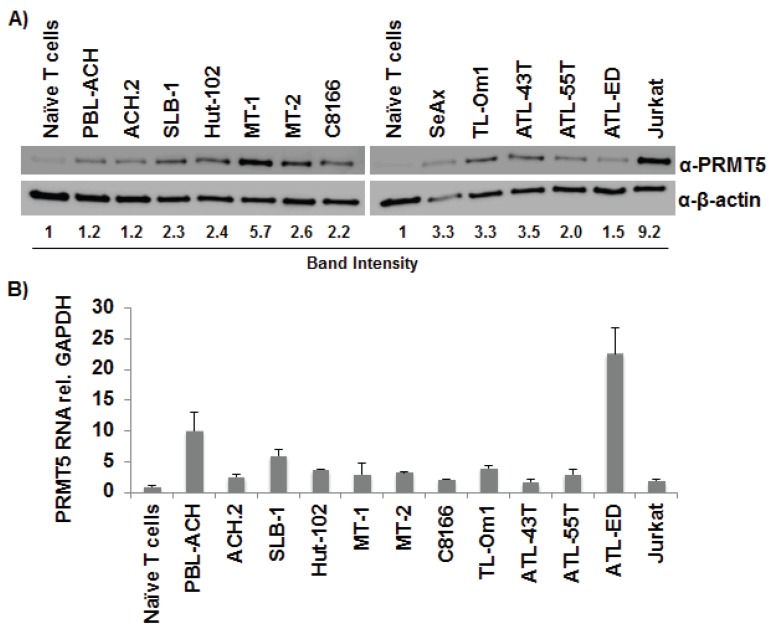

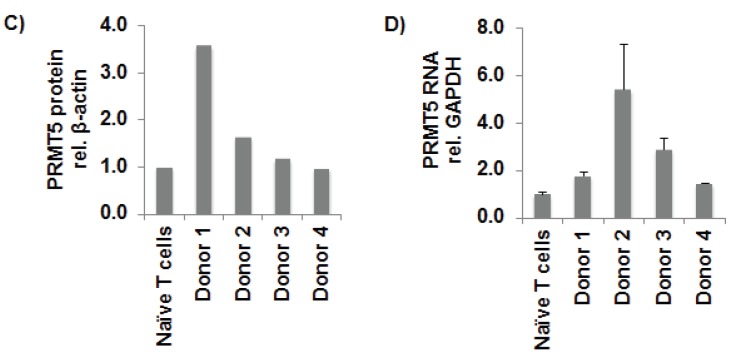
PRMT5 was upregulated in T-cell leukemia/lymphoma cells. (**A**) Total cell lysates of HTLV-1-transformed cell lines (PBL-ACH, ACH.2, SLB-1, Hut-102, MT-1, MT-2, C8166), ATL-derived cell lines (TL-Om1, ATL-43T, ATL-55T, ATL-ED), HTLV-1-negative transformed cell lines (SeAx, Jurkat), and naïve T-cells were subjected to immunoblot analysis to compare the levels of endogenous PRMT5 expression. β-Actin expression was used as a loading control. The amount of PRMT5 in each cell line was measured relative to β-actin; the level of PRMT5 expression obtained with naïve T-cells was set at 1; (**B**) Quantitative RT-PCR for PRMT5 and GAPDH was performed on mRNA isolated from cells in panel A. Total PRMT5 mRNA level was determined using the ΔΔCt method [47] and normalized to relative GAPDH levels. Data are presented in histogram form with means and standard deviations from triplicate experiments; (**C**) Lysates of ATLL PBMCs from four independent donors and naïve T-cells were subjected to immunoblot analysis to compare endogenous PRMT5 protein expression levels. β-Actin expression was used as a loading control. The amount of PRMT5 in each condition was measured relative to β-actin and depicted in histogram form; the level of expression obtained with naïve T-cells was set to 1. Each sample was measured in duplicate; (**D**) Quantitative RT-PCR for PRMT5 and GAPDH was performed on mRNA isolated from cells in panel C. Total PRMT5 mRNA level was determined using the ΔΔCt method and normalized to relative GAPDH levels. Data are presented in histogram form with means and standard deviations from triplicate experiments.

Recently, PRMT5 over-expression was identified to be involved in the pathogenesis of hematologic (lymphoma) and solid tumors (melanoma, astrocytomas) [27,28,29,30,31,32]. To determine whether PRMT5 is important to HTLV-1 biology and pathogenesis, we first examined the levels of PRMT5 protein (Figure 1A) and RNA (Figure 1B) in a wide variety of T lymphocytic leukemia/lymphoma cells, including HTLV-1-transformed T-cell lines (PBL-ACH, ACH.2, SLB-1, Hut-102, MT-1, MT-2, C8166), ATL-derived T-cell lines (TL-Om1, ATL-43T, ATL-55T, ATL-ED), HTLV-1-negative T-cell lines (SeAx, Jurkat), and naïve primary T-cells. Both PRMT5 protein and RNA were upregulated in all T-cell leukemia/lymphoma cell lines compared to naïve T-cells. Interestingly, although protein and RNA levels were upregulated, PRMT5 RNA was not directly correlated to PRMT5 protein levels, which suggested a post-transcriptional method of regulation. PRMT5 protein (Figure 1C) and RNA (Figure 1D) were also upregulated in 3 of 4 and 4 of 4 PBMC samples from ATLL patients, respectively, relative to HTLV-1-negative naïve T-cells.

### 3.2. PRMT5 Levels Were Elevated during HTLV-1-Mediated Cellular Transformation

**Figure 2:**
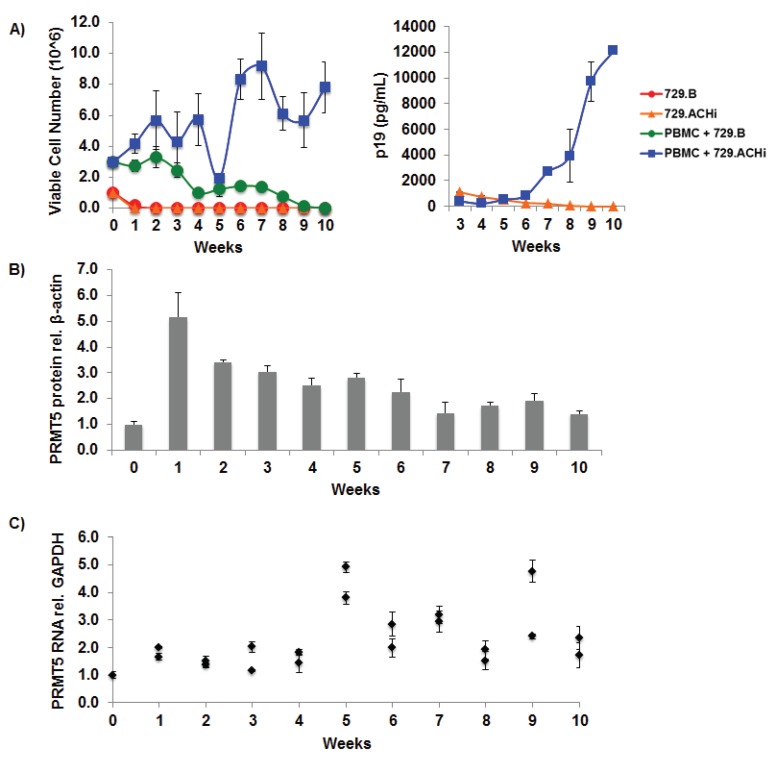
PRMT5 levels were elevated during HTLV-1-mediated cellular transformation. Freshly isolated PBMCs (2 × 10^6^ cells) were co-cultivated with 10^6^ lethally irradiated 729.B uninfected parental or 729.ACH HTLV-1-producer cells in 24-well plates. (**A**) A growth curve showing the immortalization process as measured by T-cell number and ELISA data showing p19 Gag protein production at weekly intervals are presented. Means and standard deviations of data from each time point were determined from four random independent wells; Cells were also collected at weekly intervals and analyzed by immunoblotting for PRMT5 protein expression (**B**); and qRT-PCR analysis for PRMT5 RNA levels (**C**). PRMT5 levels are shown relative to an internal control (β-actin, GAPDH) for each time point. Resting PBMC PRMT5 levels were set to 1. Means and standard deviations of data from each time point were determined from two random independent wells.

We next determined whether PRMT5 becomes dysregulated and over-expressed during HTLV-1-driven T-cell transformation. Freshly isolated human PBMCs co-cultured with lethally irradiated HTLV-1 producer cells (729.ACHi) in the presence of 10 U/mL of human IL-2 showed progressive growth consistent with HTLV-1 immortalization (Figure 2A, left panel). As a control, PBMCs co-cultured with lethally irradiated 729.B (HTLV-1-negative parental line) were unable to sustain progressive growth. We also detected continuous accumulation of p19 Gag in the culture supernatants of PBMCs co-cultured with 729.ACHi cells, which indicated viral replication and virion production; as expected, irradiated HTLV-1 producer cells alone failed to grow or produce p19 over time (Figure 2A, right panel). We examined PRMT5 protein (Figure 2B) and RNA (Figure 2C) levels throughout the 10-week *in vitro* transformation assay. Protein and RNA were isolated from two independent wells of cells at weekly time points. Our data revealed that PRMT5 protein and RNA were upregulated at each time point, to varying degrees, throughout the transformation assay.

### 3.3. Loss of Endogenous PRMT5 Increased HTLV-1 Gene Expression

**Figure 3:**
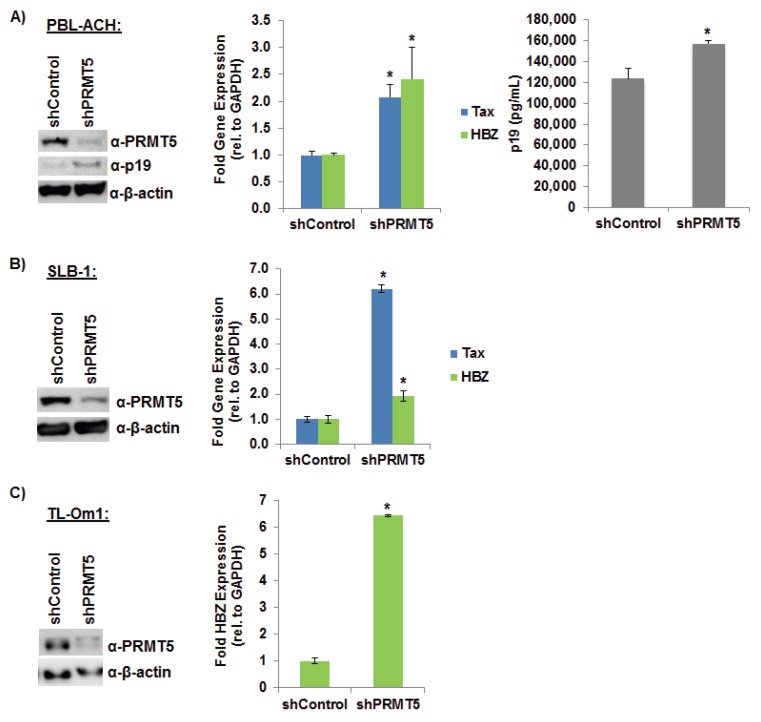
Loss of endogenous PRMT5 increased HTLV-1 gene expression. (**A**) PBL-ACH cells (HTLV-1-transformed) were infected with a pool of five different lentiviral vectors directed against PRMT5, or control shRNAs. The cells were selected for 7 days using puromycin. Immunoblot analysis was performed to compare the levels of PRMT5, p19 (Gag), and β-actin (loading control) in each condition (left panel). Quantitative RT-PCR (middle panel) for Tax, HBZ, and GAPDH was performed on mRNA isolated from shControl and shPRMT5 cells. Transcript levels were determined using the ΔΔCt method and normalized to relative GAPDH levels. Levels of Tax and HBZ relative to GAPDH in shControl cells were set to 1. Data are presented in histogram form with means and standard deviations from triplicate experiments. HTLV-1 gene expression was quantified by the detection of the p19 Gag protein in the culture supernatant using ELISA (right panel); (**B**) SLB-1 cells (HTLV-1-transformed) were infected with a pool of five different lentiviral vectors directed against PRMT5 or control shRNAs. The cells were selected for 7 days using puromycin. Immunoblot analysis was performed to compare the levels of PRMT5 and β-actin protein (loading control) in each condition (left panel). Quantitative RT-PCR for Tax, HBZ, and GAPDH was performed on mRNA isolated from shControl and shPRMT5 cells (right panel). Transcript levels were determined using the ΔΔCt method and normalized to relative GAPDH levels. Data are presented in histogram form with means and standard deviations from triplicate experiments. Levels of Tax and HBZ relative to GAPDH in shControl cells were set to 1; (**C**) TL-Om1 cells (ATL-derived, Tax-negative) were infected with a pool of five different lentiviral vectors directed against PRMT5, or control shRNAs. The cells were selected for 7 days using puromycin. Immunoblot analysis was performed to compare the levels of PRMT5 and β-actin protein (loading control) in each condition (left panel). Quantitative RT-PCR for HBZ and GAPDH was performed on mRNA isolated from shControl and shPRMT5 cells (right panel). HBZ transcript level was determined using the ΔΔCt method and normalized to relative GAPDH levels. Data are presented in histogram form with means and standard deviations from triplicate experiments. Levels of HBZ relative to GAPDH in shControl cells were set to 1. Student’s *t* test was performed to determine significant differences in viral transcript levels between shControl and shPRMT5 cells; *p* < 0.05 (*).

To determine whether PRMT5 over-expression is a marker for T-cell transformation and/or contributes to the process of HTLV-1 transformation, we utilized shRNA vectors to knockdown PRMT5 expression in three different HTLV-1-transformed cell lines. As shown in Figure 3A, shRNA-mediated knockdown of endogenous PRMT5 expression in the early passage HTLV-1 immortalized T-cell line, PBL-ACH, resulted in a significant increase of viral p19 Gag protein production (left and right panel) and a significant increase in the levels of Tax and HBZ gene expression (middle panel). Knockdown of PRMT5 expression likewise significantly increased Tax and HBZ transcript levels in another HTLV-1-transformed cell line, SLB-1 (Figure 3B). Finally, knockdown of PRMT5 protein expression in the Tax-negative ATL-derived T-cell line, TL-Om1, significantly increased HBZ transcript levels (Figure 3C).

### 3.4. Selective Inhibition of PRMT5 Enhanced HTLV-1 Gene Expression

**Figure 4:**
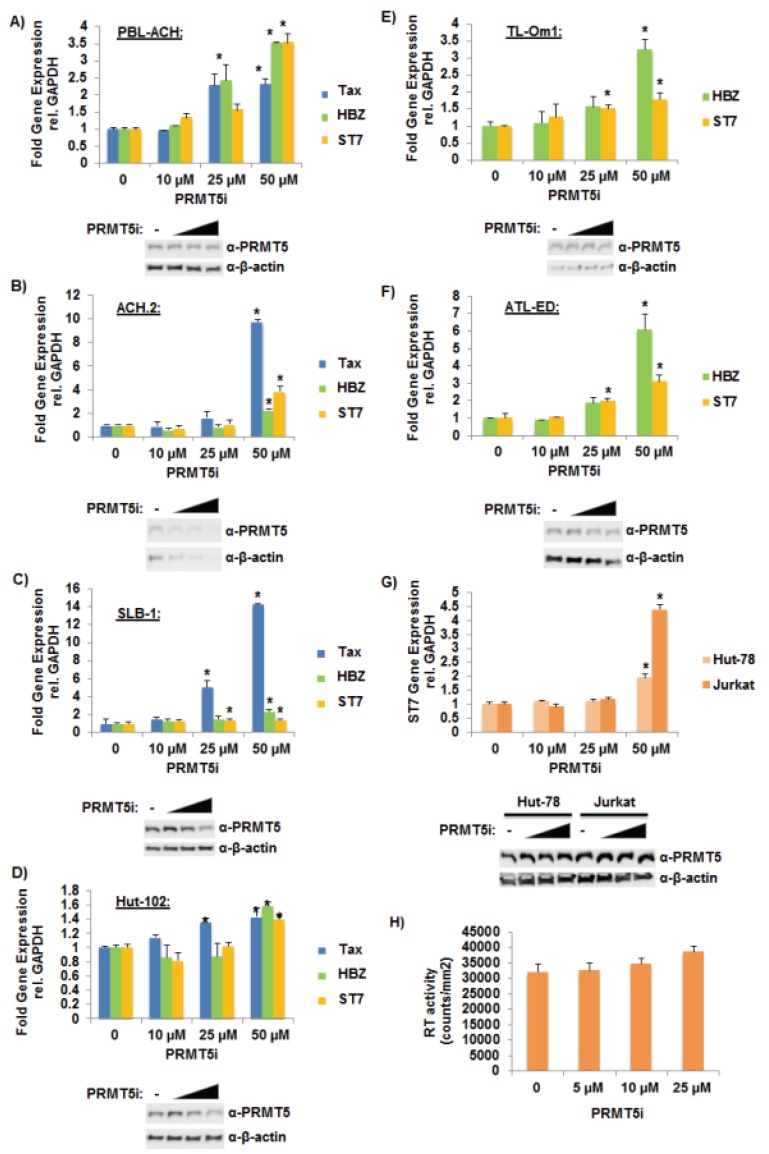
Selective inhibition of PRMT5 enhanced HTLV-1 gene expression. (**A**) PBL-ACH, (**B**) ACH.2, (**C**) SLB-1, (**D**) Hut-102, (**E**) TL-Om1, (**F**) ATL-ED, (**G**) Jurkat, and Hut-78 cells were treated with titrating amounts of PRMT5i for 48 h. (PBL-ACH, ACH.2, SLB-1, and Hut-102 cells are HTLV-1-tranformed lines; TL-Om1 and ATL-ED are ATL-derived Tax-negative lines; Jurkat and Hut-78 are HTLV-1 negative lines.) Cells were collected by slow centrifugation at 800 x*g* for 5 min. Quantitative RT-PCR for Tax (where indicated), HBZ (where indicated), ST7, and GAPDH was performed on mRNA isolated from cells in each condition. Transcript level was determined using the ΔΔCt method and normalized to relative GAPDH levels. Data are presented in histogram form with means and standard deviations from duplicate experiments; vehicle-treated cells were set to 1 (upper panel). Student’s *t* test was performed to determine significant differences in viral transcript levels between vehicle and inhibitor treated cells; *p* < 0.05 (*). Inhibitor-treated cells were also subjected to immunoblot analysis to compare the levels of endogenous PRMT5 expression. β-actin expression was used as a loading control (lower panel); (**H**) ACH-2 cells were treated with titrating amounts of PRMT5i for 48 h. Average reverse transcriptase (RT) activity of triplicate experiments is depicted.

Recently, a first-in-class, small-molecule PRMT5 inhibitor (PRMT5i) was developed [31]. This novel inhibitor selectively blocks S2Me-H4R3 (symmetric di-methylation of H4R3) but is inactive against other type I and type II PRMT enzymes, which highlights its specificity for PRMT5. To evaluate whether PRMT5 over-expression is a marker for T-cell transformation or contributes to the process of HTLV-1 transformation, we treated six different HTLV-1-transformed cell lines with titrating amounts of PRMT5i ranging from 10 μM to 50 μM. As shown in Figure 4A–D, inhibition of PRMT5 resulted in a significant increase in Tax and HBZ viral gene expression in the HTLV-1-transformed T-cell lines PBL-ACH, ACH.2 SLB-1, and Hut-102. ST7 transcript levels were measured as a control to ensure successful PRMT5 inhibition because PRMT5 was reported to repress the tumor suppressor ST7 in MCL [28]. Treatment with PRMT5i also resulted in a significant increase in HBZ transcript levels in the Tax-negative, ATL-derived T-cell lines TL-Om1 and ATL-ED (Figure 4E,F). HTLV-1-negative cell lines, Jurkat and Hut-78, were also treated with titrating amounts of PRMT5i. ST7 transcript levels were measured to ensure successful PRMT5 inhibition (Figure 4G). PRMT5 expression levels were examined and found to be relatively unchanged in all PRMT5i treated cells tested (western blot lower panels; Figure 4A–G). ACH-2 cells (HIV-1_LAV_) were also treated with titrating amounts of PRMT5i (Figure 4H) to examine if PRMT5 might regulate viral gene expression of another human retrovirus. Treatment with PRMT5i did not significantly alter the expression of HIV-1 as measured by RT activity in the cell supernatant.

### 3.5. Selective Inhibition of PRMT5 Decreased Cell Proliferation and Viability

**Figure 5:**
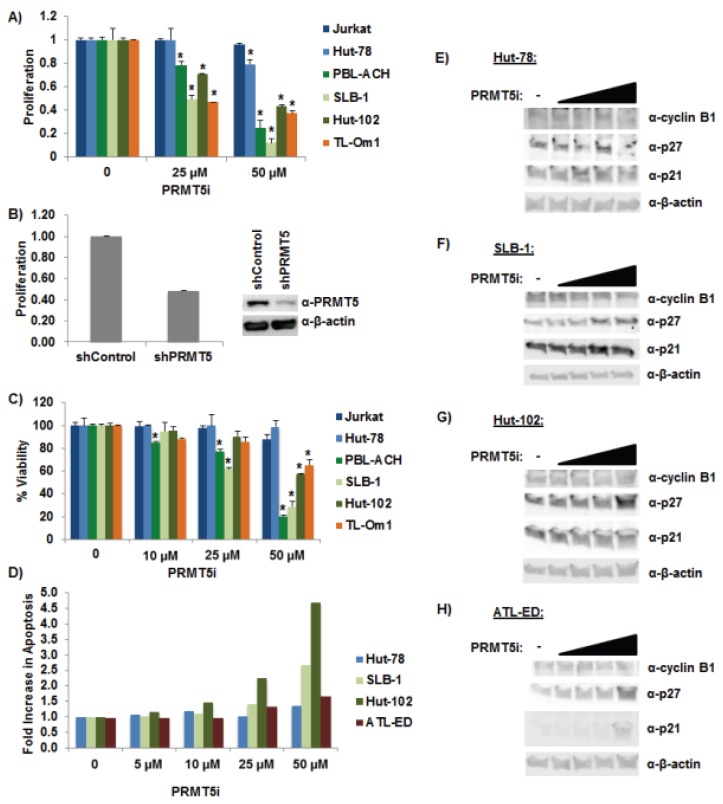
Selective inhibition of PRMT5 decreased cell proliferation and viability. (**A**) Cellular proliferation was measured using an MTS cellular proliferation assay in Jurkat, Hut-78, PBL-ACH, SLB-1, Hut-102, and TL-Om1 cell lines after 48 h of PRMT5i treatment. (Jurkat and Hut-78 are HTLV-1 negative lines; PBL-ACH, SLB-1, and Hut-102 cells are HTLV-1-tranformed lines; TL-Om1 is an ATL-derived Tax-negative line.) Data are presented in histogram form with mean and standard deviations from duplicate experiments. Vehicle treatment for each cell type was set at 1. Student’s *t* test was performed to determine significant differences in cellular proliferation between vehicle and inhibitor-treated cells; *p* < 0.05 (*); (**B**) SLB-1 cells were infected with a pool of five different lentiviral vectors directed against PRMT5 or control shRNAs. The cells were selected for 7 days using puromycin. Immunoblot analysis was performed to compare the levels of PRMT5 and β-actin protein (loading control) in each condition (right panel). Cellular proliferation was measured using an MTS cellular proliferation assay (left panel); (**C**) Cellular viability was measured using Trypan blue exclusion method in Jurkat, Hut-78, PBL-ACH, SLB-1, Hut-102, and TL-Om1 after 48 h of PRMT5i treatment. Data are presented in histogram form with means and standard deviations from duplicate experiments. Vehicle treatment for each cell type was set at 100% viability. Student’s *t* test was performed to determine significant differences in cellular viability between vehicle and inhibitor treated cells; *p* < 0.05 (*); (**D**) Cellular apoptosis was measured using a FITC Annexin V Apoptosis Detection Kit as described in the Materials and Methods. The percentage of cells undergoing apoptosis in vehicle (DMSO) treated cells was set at 1. The fold increase in apoptosis was measured in Hut-78, SLB-1, Hut-102, and ATL-ED cells in response to titrating amounts of PRMT5i after 24 h of treatment; (ATL-ED is an ATL-derived Tax-negative line.) (**E**–**H**) Immunoblot analysis was performed in Hut-78, SLB-1, Hut-102, and ATL-ED cells after 24 h of PRMT5i treatment to compare the levels of p21, p27, cyclin B1, and β-actin expression (loading control) in each condition (right panel).

In a recent report, PRMT5 was linked to proliferation in B-cell lines and MCL because knockdown of PRMT5 expression reduced cell proliferation [28]. Treatment of HTLV-1-positive T-cell lines with titrating amounts of PRMT5i or shRNA-mediated knockdown of PRMT5 resulted in a significant decrease in cellular proliferation (Figure 5A,B) and cell viability (Figure 5C). Surprisingly, the same dose of PRMT5i had minimal effects on proliferation and viability of Jurkat and Hut-78 cells (HTLV-1-negative). The level of cellular apoptosis (Figure 5D) and senescence (Figure 5E–H) was also measured in response to titrating amounts of PRMT5i treatment. The number of apoptotic cells was increased in all cell lines in response to PRMT5i treatment; however, the amount of apoptotic cells in HTLV-1-transformed lines was higher. We found a slight increase in the level of cellular senescence (as measured by increased p21 and p27 levels and decreased cyclin B1 levels) in response to PRMT5i in the HTLV-1-positive cell lines examined.

### 3.6. PRMT5 Negatively Regulated HTLV-1 Gene Expression

**Figure 6:**
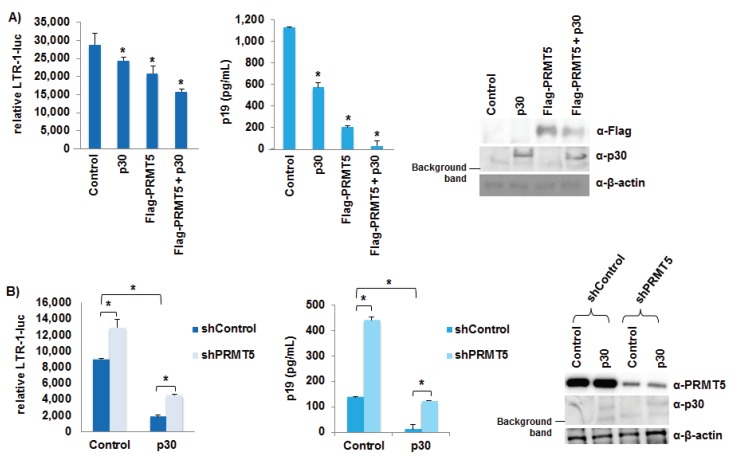
PRMT5 negatively regulated HTLV-1 gene expression. (**A**) HEK293T cells were transfected with 20 ng TK-renilla, 100 ng LTR-1-luciferase reporter, 1 μg ACHneo, 500 ng flag-PRMT5, and 500 ng p30 expression plasmid as indicated. At 48 h post-transfection, cell lysates were collected and luciferase levels measured; relative luciferase activity for each condition is shown (left panel). The decreases in relative LTR-1-luc activity compared to control were significant (*p* < 0.05 (*)). HTLV-1 gene expression was quantified by the detection of the p19 Gag protein in the culture supernatant of each condition using ELISA (middle panel). The decreases in p19 Gag levels compared to control were significant (*p* < 0.05 (*)). Immunoblot analysis was performed to compare the levels of PRMT5 (Flag), p30, and β-actin (loading control) in each condition (right panel); (**B**) HEK293T cells were infected with a pool of five different lentiviral vectors directed against PRMT5 or control shRNAs. The cells were selected for 7 days using puromycin. HEK293T shControl and shPRMT5 cells were then transfected with 20 ng TK-renilla, 100 ng LTR-1-luciferase reporter, 1 μg ACHneo, and 500 ng p30 expression plasmid as indicated. Forty-eight hours post-transfection, cell lysates were collected, and luciferase levels measured; relative luciferase activity for each condition is shown (left panel). The differences in relative LTR-1-luc activity were significant (*p* < 0.05 (*)). HTLV-1 gene expression was quantified by the detection of the p19 Gag protein in the culture supernatant of each condition using ELISA (middle panel). The differences in p19 Gag levels were significant (*p* < 0.05 (*)). Immunoblot analysis was performed to compare the levels of endogenous PRMT5, p30, and β-actin (loading control) in each condition (right panel).

A recent report identified PRMT5 as a binding partner of the HTLV-1 accessory protein p30, a known negative regulator of HTLV-1 gene expression [37]. To investigate the effect(s) of exogenous PRMT5 on p30, HEK293T cells were transfected with a LTR-1-luciferase reporter vector (LTR-1-luc), TK-renilla control, the ACHneo proviral clone, a flag-tagged PRMT5 expression vector, and a p30 expression vector as indicated (Figure 6A). Luciferase activity was measured after 48 h. The luciferase activity of the empty control reflected the amount of Tax and therefore, was a measure of transcription from the provirus. In the presence of either exogenous p30 or PRMT5, LTR-1-luciferase reporter was significantly repressed (left panel). However, in the presence of both p30 and PRMT5, there was an additive effect on LTR-1-luciferase repression. The amount of viral p19 Gag protein present in the culture supernatant of each condition also was examined using ELISA, which provided another method to quantify HTLV-1 gene expression (middle panel). Similar to the luciferase results, p30 and PRMT5 individually repressed viral p19 Gag production, and the presence of both p30 and PRMT5 repressed viral gene transcription further. We next asked whether PRMT5 was required for p30 function by transducing HEK293T cells with shRNA vectors directed against PRMT5 or a scramble control (Figure 6B). Scramble and shPRMT5 HEK293T cell lines were selected for 7 days using puromycin to ensure sufficient knockdown of endogenous PRMT5. After selection, each cell line was transfected with LTR-1-luc, TK-renilla control, the ACHneo proviral clone, and a p30 expression vector as indicated. Luciferase activity was measured after 48 h. Knockdown of endogenous PRMT5 significantly enhanced viral transcription, as measured by LTR-1-luciferase activity (left panel) and p19 Gag ELISA (middle panel). In addition, reduced levels of PRMT5 did not significantly affect the ability of p30 to repress viral transcription.

### 3.7. PRMT5 Did Not Affect Tax Transcriptional Function

**Figure 7:**
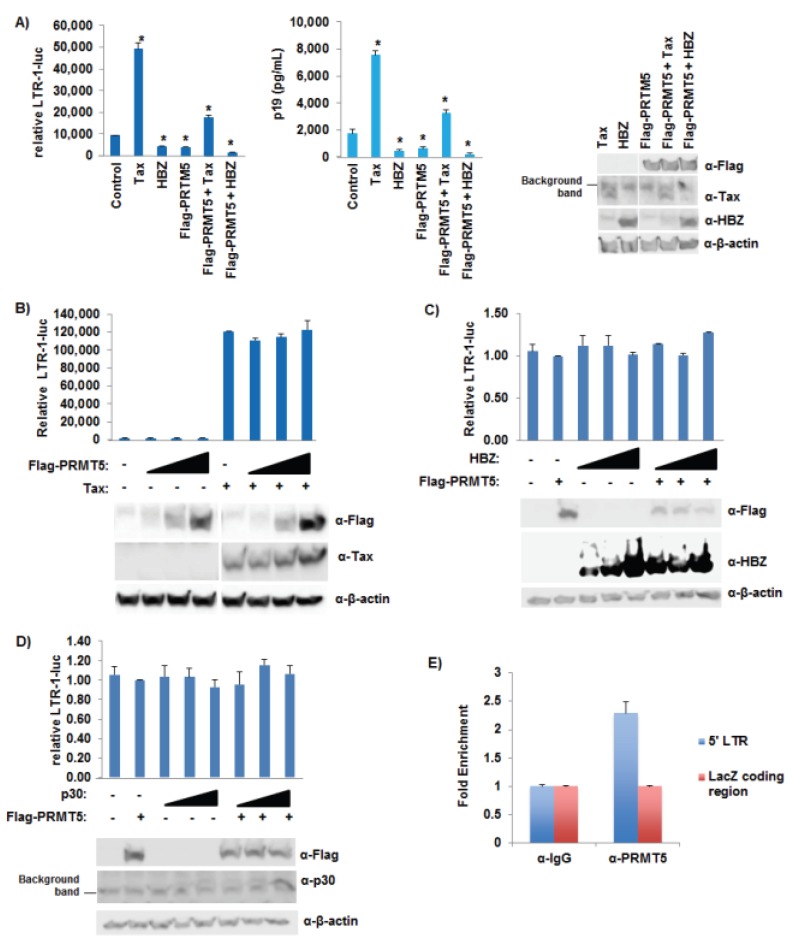
PRMT5 did not affect Tax transcriptional function. (**A**) HEK293T cells were transfected with 20 ng TK-renilla, 100 ng LTR-1-luciferase reporter, 1 μg ACHneo, 100 ng S-tag-Tax, 500 ng S-tag-HBZ, and 500 ng flag-PRMT5 as indicated. Forty-eight hours post-transfection, cell lysates were collected and luciferase levels measured; relative luciferase activity for each condition is shown (left panel). The differences in relative LTR-1-luc activity compared to control were significant (*p* < 0.05 (*)). HTLV-1 gene expression was quantified by the detection of the p19 Gag protein in the culture supernatant of each condition using ELISA (middle panel). The differences in p19 Gag levels compared to control were significant (*p* < 0.05 (*)). Immunoblot analysis was performed to compare the levels of PRMT5 (Flag), Tax, HBZ, and β-actin (loading control) in each condition (right panel). All samples were analyzed on the same nitrocellulose membrane; (**B**) HEK293T cells were transfected with 20 ng TK-renilla, 100 ng LTR-1-luciferase reporter, 100 ng S-tag-Tax, and titrating amounts of flag-PRMT5 as indicated. Forty-eight hours post-transfection, cell lysates were collected, and luciferase levels measured; relative luciferase activity for each condition is shown (upper panel). Immunoblot analysis was performed to compare the levels of PRMT5 (Flag), Tax, and β-actin (loading control) in each condition (lower panel). All samples were analyzed on the same nitrocellulose membrane; (**C**) HEK293T cells were transfected with 20 ng TK-renilla, 100 ng LTR-1-luciferase reporter, 500 ng flag-PRMT5, and titrating amounts of HBZ as indicated. Forty-eight hours post-transfection, cell lysates were collected and luciferase levels measured; relative luciferase activity for each condition is shown (upper panel). Immunoblot analysis was performed to compare the levels of PRMT5 (Flag), HBZ, and β-actin (loading control) in each condition (lower panel); (**D**) HEK293T cells were transfected with 20 ng TK-renilla, 100 ng LTR-1-luciferase reporter, 500 ng flag-PRMT5, and titrating amounts of p30 as indicated. Forty-eight hours post-transfection, cell lysates were collected and luciferase levels measured; relative luciferase activity for each condition is shown (upper panel). Immunoblot analysis was performed to compare the levels of PRMT5 (Flag), p30, and β-actin (loading control) in each condition (lower panel); (**E**) ChIP assays were performed on cross-linked chromatin from pA-18G-BHK-21 cells using either IgG or PRMT5 antibodies, and the retained DNA was amplified using LTR or LacZ coding region-specific primers. Fold-enrichment with each antibody was calculated relative to the IgG sample. Each ChIP experiment was repeated twice in duplicate.

We next asked what effect PRMT5 had on Tax and HBZ transcriptional activity. HEK293T cells were transfected with LTR-1-luc, TK-renilla control, the ACHneo proviral clone, flag-PRMT5 expression vector, S-tag-Tax expression vector, and S-tag-HBZ expression vector as indicated (Figure 7A). Tax activated transcription while HBZ repressed Tax-mediated transcriptional activation (left and middle panels), as expected [17,18,19]. In the presence of exogenous PRMT5, Tax transcriptional activity and HBZ-mediated repression of Tax transcriptional activity were decreased. To determine if PRMT5 was able to specifically repress Tax in the absence of other viral genes, HEK293T cells were transfected with LTR-1-luc, TK-renilla control, flag-PRMT5 expression vector, and an S-tag-Tax expression vector (Figure 7B). Tax was able to activate LTR-1-luciferase activity, while PRMT5 had no effect in the presence or absence of Tax on LTR-1 luciferase activity. Our results suggested that PRMT5 requires the HTLV-1 proviral DNA to suppress Tax-induced LTR activation. To examine if viral factors other than Tax are implicated in suppression of viral transcription by PRMT5, we examined the effect of HBZ or p30 with PRMT5 on LTR-1 luciferase activity. HEK293T cells were transfected with LTR-1-luc, TK-renilla control, flag-PRMT5 expression vector, and an S-tag-HBZ or p30 expression vector (Figure 7C,D). Neither HBZ nor p30 activated or repressed LTR-1-luciferase activity in the absence of Tax, as expected [17,18,19,20,21,22,41,42]. Also, PRMT5 had no effect in the presence or absence of HBZ or p30 on LTR-1-luciferase activity. To determine if PRMT5 associated with the viral LTR promoters *in vivo*, we performed ChIP assays using pA-18G-BHK-21 cells. The Syrian Hamster kidney cell line was stably transfected with a plasmid vector containing the HTLV-1-LTR promoter that drives expression of lacZ [46]. pA-18G-BHK-21 cells were transfected with ACHneo proviral DNA and flag-PRMT5 expression vector (Figure 7E). PRMT5 was associated with the viral LTR, but not the downstream LacZ-coding region (negative control).

## 4. Discussion

HTLV-1 is a tumorigenic retrovirus and the causative infectious agent of ATLL, an extremely aggressive and fatal disease of CD4+ T-cells [2,3,4]. In culture, HTLV-1 can effectively immortalize and eventually transform primary human T-cells. However, in infected individuals, the incidence of disease is only 2%–6% [7] after an extensive clinical latency period. Evidence suggests both genetic and epigenetic changes in the cellular environment that accumulate over time contribute to the development of ATLL [23]. While many aspects of HTLV-1 biology have been revealed, the detailed mechanisms of ATLL development remain poorly defined. Recently, the cellular protein PRMT5 has been shown to play a critical role in EBV-driven B-cell transformation as well as the pathogenesis of many types of hematologic and solid tumors [27,28,29,30,31,32]. We hypothesized that PRMT5 could be important in HTLV-1-mediated cellular transformation and in regulation of viral replication. Given the development of a novel small molecular inhibitor (PRMT5i) [31], identification of PRMT5 as a factor during HTLV-1 transformation will provide valuable insights into improved strategies to treat patients with ATLL.

To determine the importance of PRMT5 in HTLV-1-infected cells, we first examined the expression level of endogenous PRMT5 in a variety of HTLV-1-transformed, ATLL-derived, and HTLV-1-negative T-cells lines (Figure 1A). PRMT5 proteins levels were upregulated in HTLV-1-positive cells, but also in all transformed T-cell lines, regardless of origin. This is not surprising given that PRMT5 over-expression has recently been identified in lung carcinoma, glioblastoma, B-cell lymphoma, mantle cell lymphoma, and melanoma, to name just a few [28,31,48,49,50]. It appears that PRMT5 over-expression is a hallmark of most transformed cells, not specifically HTLV-1-transformed cells. Previous work by Pal *et al.* found decreased PRMT5 mRNA levels in mantle cell lymphoma cell lines despite abundant PRMT5 protein over-expression [28]. In this instance, the increase in PRMT5 protein was not due to an increase in mRNA levels, but instead was due to a decrease in the inhibitory miRNAs miR-92b and miR-96, which allowed for enhanced PRMT5 translation. Conversely, a recent report by Shilo *et al.* found both PRMT5 protein and mRNA were upregulated in lung tumors [48]. We examined the level of PRMT5 mRNA in a variety of HTLV-1-transformed, ATLL-derived, and HTLV-1-negative T-cell lines and found that the PRMT5 mRNA level was increased in every transformed cell line relative to naïve T-cells (Figure 1B). However, the increase in PRMT5 mRNA did not directly correlate with the level of PRMT5 protein expression, which suggested some degree of post-transcriptional regulation. Because these experiments were conducted in cell lines grown *in vitro*, we also examined the level of PRMT5 protein and RNA in total PBMCs isolated from ATLL patients (Figure 1C,D). Both PRMT5 protein and RNA were upregulated in a majority of ATLL patient samples. The increased level of PRMT5 RNA and protein expression in patient PBMCs was not as prominent as what was found in transformed cell lines, likely due to the use of total PBMCs, which contain a mixture of normal and leukemic cells.

HTLV-1 infection of CD4+ T-cells does not always lead to transformation. A delicate balance must be achieved between viral gene expression and certain genetic and epigenetic events to result in transformation. Using a long-term immortalization co-culture assay, we found both PRMT5 protein and RNA were upregulated throughout the immortalization process (Figure 2). It is important to note the producer cells were lethally irradiated, and although there was some residual p19 Gag detected in the supernatant, the producer cells were dead by week 1. Since only a portion of the total co-culture assay was tested, the levels of both protein and RNA fluctuated from week to week; however, the overall trend showed that PRMT5 was upregulated.

Regulation of viral gene expression early after infection is highly relevant for successful transformation; for example, too much Tax expression can cause a phenomenon known as Tax-induced senescence (TIS) [51]. Using shRNA vectors directed against PRMT5, we found that knockdown of PRMT5 enhanced HTLV-1 viral gene expression and decreased cellular proliferation in HTLV-1-infected cell lines (Figure 3 and Figure 5B). Given the importance of PRMT5 in cellular proliferation, long-term stable cell lines were difficult to create. Thus, we transduced cells and selected with drug for less than two weeks, which provided the added benefit of less antigenic drift within the cell population over time. Similar results were obtained using a novel, small molecule inhibitor of PRMT5 (Figure 4A–F). Because Tax expression is lost in a majority of ATLL-transformed cells and only HBZ is expressed in every cell, we included the Tax-negative ATLL transformed cell lines, TL-Om1 and ATL-ED, in our studies. PRMT5 knockdown and inhibition enhanced HBZ expression in ATLL transformed cell lines. Of interest, PRMT5i did not affect HIV-1 gene expression, which suggested that PRMT5 was not a global repressor of all retrovirus gene expression (Figure 4G). Because Tax and HBZ are driven from separate viral promoters (5' LTR and 3' LTR opposite strand, respectively), this finding would suggest that PRMT5 is a global repressor of HTLV-1 transcription. In support of this hypothesis, we found PRMT5 associated with the viral LTR using ChIP analysis (Figure 7E).

Using reporter gene assays, we found PRMT5 inhibited HTLV-1 gene transcription, but not Tax protein specifically (Figure 6 and Figure 7A,B). We also found LTR promoter activation was unaffected by PRMT5 (with or without viral accessory proteins HBZ or p30) in the absence of the proviral genome (Figure 7B–D). This result was not surprising since one of the functions of HBZ is to repress Tax-mediated transcriptional activation of the viral LTR and the function of p30 is to retain unspliced *tax/rex* mRNA in the nucleus. Taken together, these results suggested that other viral proteins were required for the repressive effects of PRMT5, and/or PRMT5 affected a cellular transcription factor responsible for activating viral transcription. Although not required for the repressive effects of p30 on viral gene expression, we did find PRMT5 and p30 had additive repressive effects on viral transcription, which adds yet another level of regulation to HTLV-1 gene expression. The roles of additional PRMT5 interacting partners, such as MEP50, in PRMT5-mediated HTLV-1 gene regulation are also a possibility to explore in the future. MEP50 is a WD-40 repeat protein and a common PRMT5 cofactor, likely present in most PRMT5-containing complexes *in vivo* [52]. Phosphorylation of MEP50 by Cdk4 alters the activity and targeting of the PRMT5 protein in cells [53].

Importantly, we found PRMT5i treatment or shRNA-mediated knockdown of PRMT5 in HTLV-1-positive cell lines caused a decrease in cell proliferation compared to HTLV-1-negative cell lines (Figure 5A,B). Furthermore, PRMT5i was selectively toxic to HTLV-1-positive cell lines (Figure 5C). These results suggested that HTLV-1-positive cells rely strongly on PRMT5 for cellular growth and survival. Treatment with PRMT5i induced cellular apoptosis to some degree in all cell lines (Figure 5D). Interestingly, HTLV-1-transformed cell lines underwent noticeably more apoptosis than either the HTLV-1-negative or the ATL-derived cell lines. Previous reports have found that aberrant expression of Tax protein can lead to TIS in cells [51]. We did observe a slight increase in cellular senescence in response to PRMT5i in the HTLV-1-positive cell lines tested, including Tax-expressing HTLV-1-transformed cells and Tax-negative ATL-derived cells (Figure 5E–H). We would predict an increase in cellular senescence in the HTLV-1-transformed cell lines, as they are the only Tax-expressing lines. However, these cell lines also express HBZ, which has been reported to repress TIS. Another possibility is the level of Tax expression induced in response to PRMT5i in our cell lines was not substantial enough to elicit a measurable increase in cellular senescence. In summary, our study highlighted the significance of PRMT5 in HTLV-1-mediated cellular transformation and its importance as a target for the newly developed PRMT5i, presenting a viable strategy for treatment of ATLL.

## References

[1] Proietti F.A., Carneiro-Proietti A.B., Catalan-Soares B.C., Murphy E.L. (2005). Global epidemiology of HTLV-I infection and associated diseases. Oncogene.

[2] Poiesz B.J., Ruscetti F.W., Gazdar A.F., Bunn P.A., Minna J.D., Gallo R.C. (1980). Detection and isolation of type C retrovirus particles from fresh and cultured lymphocytes of a patient with cutaneous T-cell lymphoma. Proc. Natl. Acad. Sci. USA.

[3] Yoshida M., Miyoshi I., Hinuma Y. (1982). Isolation and characterization of retrovirus from cell lines of human adult T-cell leukemia and its implication in the disease. Proc. Natl. Acad. Sci. USA.

[4] Hinuma Y., Nagata K., Hanaoka M., Nakai M., Matsumoto T., Kinoshita K.-I., Shirakawa S., Miyoshi I. (1981). Adult T-cell leukemia: Antigen in an ATL cell line and detection of antibodies to the antigen in human sera. Proc. Natl. Acad. Sci. USA.

[5] Osame M., Izumo S., Igata A., Matsumoto M., Matsumoto T., Sonoda S., Tara M., Shibata Y. (1986). Blood transfusion with HTLV-I associated myelopathy. Lancet.

[6] Gessain A., Barin F., Vernant J.C., Gout O., Maurs L., Calender A., de The G. (1985). Antibodies to human T-lymphotropic virus type-I in patients with tropical spastic paraparesis. Lancet.

[7] Taylor G.P. (2015). Editorial commentary: Human T-cell lymphotropic virus type 1 (HTLV-1) and HTLV-1-associated myelopathy/tropical spastic paraparesis. Clin. Infect. Dis..

[8] Utsunomiya A., Choi I., Chihara D., Seto M. (2015). Recent advances in the treatment of adult T-cell leukemia-lymphomas. Cancer Sci..

[9] Cheng H., Ren T., Sun S.C. (2012). New insight into the oncogenic mechanism of the retroviral oncoprotein Tax. Protein Cell.

[10] Matsuoka M., Green P.L. (2009). The HBZ gene, a key player in HTLV-1 pathogenesis. Retrovirology.

[11] Grassmann R., Aboud M., Jeang K.T. (2005). Molecular mechanisms of cellular transformation by HTLV-1 Tax. Oncogene.

[12] Bex F., Gaynor R.B. (1998). Regulation of gene expression by HTLV-I Tax protein. Methods.

[13] Arima N., Tei C. (2001). HTLV-I Tax related dysfunction of cell cycle regulators and oncogenesis of adult T cell leukemia. Leuk. Lymphoma.

[14] Satou Y., Yasunaga J., Yoshida M., Matsuoka M. (2006). HTLV-I basic leucine zipper factor gene mRNA supports proliferation of adult t cell leukemia cells. Proc. Natl. Acad. Sci. USA.

[15] Arnold J., Zimmerman B., Li M., Lairmore M.D., Green P.L. (2008). Human T-cell leukemia virus type-1 antisense-encoded gene, HBZ, promotes T lymphocyte proliferation. Blood.

[16] Arnold J., Yamamoto B., Li M., Phipps A.J., Younis I., Lairmore M.D., Green P.L. (2006). Enhancement of infectivity and persistence *in vivo* by HBZ, a natural antisense coded protein of HTLV-1. Blood.

[17] Clerc I., Polakowski N., Andre-Arpin C., Cook P., Barbeau B., Mesnard J.M., Lemasson I. (2008). An interaction between the human T cell leukemia virus type 1 basic leucine zipper factor (HBZ) and the KIX domain of p300/CBP contributes to the down-regulation of Tax-dependent viral transcription by HBZ. J. Biol. Chem..

[18] Gaudray G., Gachon F., Basbous J., Biard-Piechaczyk M., Devaux C., Mesnard J. (2002). The complementary strand of the human T-cell leukemia virus type 1 RNA genome encodes a bZIP transcription factor that down-regulates viral transcription. J. Virol..

[19] Lemasson I., Lewis M.R., Polakowski N., Hivin P., Cavanagh M.H., Thebault S., Barbeau B., Nyborg J.K., Mesnard J.M. (2007). Human T-cell leukemia virus type 1 (HTLV-1) bZIP protein interacts with the cellular transcription factor CREB to inhibit HTLV-1 transcription. J. Virol..

[20] Thebault S., Basbous J., Hivin P., Devaux C., Mesnard J.M. (2004). HBZ interacts with JunD and stimulates its transcriptional activity. FEBS Lett..

[21] Basbous J., Arpin C., Gaudray G., Piechaczyk M., Devaux C., Mesnard J. (2003). HBZ factor of HTLV-1 dimerizes with transcription factors JunB and c-Jun and modulates their transcriptional activity. J. Biol. Chem..

[22] Matsumoto J., Ohshima T., Isono O., Shimotohno K. (2005). HTLV-1 HBZ suppresses AP-1 activity by impairing both the DNA-binding ability and the stability of c-Jun protein. Oncogene.

[23] Matsuoka M., Jeang K.T. (2007). Human T-cell leukaemia virus type 1 (HTLV-1) infectivity and cellular transformation. Nat. Rev. Cancer.

[24] Egger G., Liang G., Aparicio A., Jones P.A. (2004). Epigenetics in human disease and prospects for epigenetic therapy. Nature.

[25] Ganesan A., Nolan L., Crabb S.J., Packham G. (2009). Epigenetic therapy: Histone acetylation, DNA methylation and anti-cancer drug discovery. Curr. Cancer Drug Targets.

[26] Poke F.S., Qadi A., Holloway A.F. (2010). Reversing aberrant methylation patterns in cancer. Curr. Med. Chem..

[27] Majumder S., Alinari L., Roy S., Miller T., Datta J., Sif S., Baiocchi R., Jacob S.T. (2010). Methylation of histone H3 and H4 by PRMT5 regulates ribosomal RNA gene transcription. J. Cell. Biochem..

[28] Pal S., Baiocchi R.A., Byrd J.C., Grever M.R., Jacob S.T., Sif S. (2007). Low levels of miR-92b/96 induce PRMT5 translation and H3R8/H4R3 methylation in mantle cell lymphoma. EMBO J..

[29] Pal S., Vishwanath S.N., Erdjument-Bromage H., Tempst P., Sif S. (2004). Human SWI/SNF-associated PRMT5 methylates histone H3 arginine 8 and negatively regulates expression of ST7 and NM23 tumor suppressor genes. Mol. Cell. Biol..

[30] Wang L., Pal S., Sif S. (2008). Protein arginine methyltransferase 5 suppresses the transcription of the RB family of tumor suppressors in leukemia and lymphoma cells. Mol. Cell. Biol..

[31] Alinari L., Mahasenan K.V., Yan F., Karkhanis V., Chung J.H., Smith E.M., Quinion C., Smith P.L., Kim L., Patton J.T. (2015). Selective inhibition of protein arginine methyltransferase 5 blocks initiation and maintenance of B-cell transformation. Blood.

[32] Chung J., Karkhanis V., Tae S., Yan F., Smith P., Ayers L.W., Agostinelli C., Pileri S., Denis G.V., Baiocchi R.A. (2013). Protein arginine methyltransferase 5 (PRMT5) inhibition induces lymphoma cell death through reactivation of the retinoblastoma tumor suppressor pathway and polycomb repressor complex 2 (PRC2) silencing. J. Biol. Chem..

[33] Karkhanis V., Hu Y.J., Baiocchi R.A., Imbalzano A.N., Sif S. (2011). Versatility of PRMT5-induced methylation in growth control and development. Trends Biochem. Sci..

[34] Rank G., Cerruti L., Simpson R.J., Moritz R.L., Jane S.M., Zhao Q. (2010). Identification of a PRMT5-dependent repressor complex linked to silencing of human fetal globin gene expression. Blood.

[35] Kim C., Lim Y., Yoo B.C., Won N.H., Kim S., Kim G. (2010). Regulation of post-translational protein arginine methylation during HeLa cell cycle. Biochim. Biophys. Acta.

[36] Ancelin K., Lange U.C., Hajkova P., Schneider R., Bannister A.J., Kouzarides T., Surani M.A. (2006). Blimp1 associates with Prmt5 and directs histone arginine methylation in mouse germ cells. Nat. Cell Biol..

[37] Doueiri R., Anupam R., Kvaratskhelia M., Green K.B., Lairmore M.D., Green P.L. (2012). Comparative host protein interactions with HTLV-1 p30 and HTLV-2 p28: Insights into difference in pathobiology of human retroviruses. Retrovirology.

[38] Koralnik I.J., Gessain A., Klotman M.E., lo Monico A., Berneman Z.N., Franchini G. (1992). Protein isoforms encoded by the pX region of human T-cell leukemia/lymphotropic virus type I. Proc. Natl. Acad. Sci. USA.

[39] Lairmore M.D., Albrecht B., D’Souza C., Nisbet J.W., Ding W., Bartoe J.T., Green P.L., Zhang W. (2000). *In vitro* and *in vivo* functional analysis of human T cell lymphotropic virus type 1 pX open reading frames I and II. AIDS Res. Hum. Retrovir..

[40] Zhang W., Nisbet J.W., Albrecht B., Ding W., Kashanchi F., Bartoe J.T., Lairmore M.D. (2001). Human T-lymphotropic virus type 1 p30^II^ regulates gene transcription by binding CREB binding protein/p300. J. Virol..

[41] Younis I., Khair L., Dundr M., Lairmore M.D., Franchini G., Green P.L. (2004). Repression of human T-cell leukemia virus type 1 and 2 replication by a viral mRNA-encoded posttranscriptional regulator. J. Virol..

[42] Nicot C., Dundr J.M., Johnson J.R., Fullen J.R., Alonzo N., Fukumoto R., Princler G.L., Derse D., Misteli T., Franchini G. (2004). HTLV-1-encoded p30^II^ is a post-transcriptional negative regulator of viral replication. Nat. Med..

[43] Anderson M.D., Ye J., Xie L., Green P.L. (2004). Transformation studies with a human T-cell leukemia virus type 1 molecular clone. J. Virol. Methods.

[44] Li M., Green P.L. (2007). Detection and quantitation of HTLV-1 and HTLV-2 mRNA species by real-time RT-PCR. J. Virol. Methods.

[45] Green P.L., Ross T.M., Chen I.S.Y., Pettiford S. (1995). Human T-cell leukemia virus type II nucleotide sequences between *env* and the last exon of *tax/rex* are not required for viral replication or cellular transformation. J. Virol..

[46] Astier-Gin T., Portail J.P., Lafond F., Guillemain B. (1995). Identification of HTLV-I- or HTLV-II-producing cells by cocultivation with BHK-21 cells stably transfected with a LTR-lacZ gene construct. J. Virol. Methods.

[47] Livak K.J., Schmittgen T.D. (2001). Analysis of relative gene expression data using real-time quantitative PCR and the 2^−ΔΔC^_T_ method. Methods.

[48] Shilo K., Wu X., Sharma S., Welliver M., Duan W., Villalona-Calero M., Fukuoka J., Sif S., Baiocchi R., Hitchcock C.L. (2013). Cellular localization of protein arginine methyltransferase-5 correlates with grade of lung tumors. Diagn. Pathol..

[49] Yan F., Alinari L., Lustberg M.E., Martin L.K., Cordero-Nieves H.M., Banasavadi-Siddegowda Y., Virk S., Barnholtz-Sloan J., Bell E.H., Wojton J. (2014). Genetic validation of the protein arginine methyltransferase PRMT5 as a candidate therapeutic target in glioblastoma. Cancer Res..

[50] Nicholas C., Yang J., Peters S.B., Bill M.A., Baiocchi R.A., Yan F., Sif S., Tae S., Gaudio E., Wu X. (2013). PRMT5 is upregulated in malignant and metastatic melanoma and regulates expression of MITF and p27(Kip1.). PLoS ONE.

[51] Zhi H., Yang L., Kuo Y.L., Ho Y.K., Shih H.M., Giam C.Z. (2011). Nf-κb hyper-activation by HTLV-1 Tax induces cellular senescence, but can be alleviated by the viral anti-sense protein HBZ. PLoS Pathog..

[52] Krause C.D., Yang Z.H., Kim Y.S., Lee J.H., Cook J.R., Pestka S. (2007). Protein arginine methyltransferases: Evolution and assessment of their pharmacological and therapeutic potential. Pharmacol. Ther..

[53] Aggarwal P., Vaites L.P., Kim J.K., Mellert H., Gurung B., Nakagawa H., Herlyn M., Hua X., Rustgi A.K., McMahon S.B. (2010). Nuclear cyclin D1/CDK4 kinase regulates CUL4 expression and triggers neoplastic growth via activation of the PRMT5 methyltransferase. Cancer Cell.

